# Analysis of a Cooperation and Interventional Model in Humanitarian Medicine

**DOI:** 10.3389/fped.2021.705149

**Published:** 2021-10-28

**Authors:** Stefano M. Marianeschi, Nicola Uricchio, Gianandrea Bern Cerri, Simone Ghiselli, Cristina Carro, Giulia Albano, Nicola Viola

**Affiliations:** ^1^Niguarda Ca' Granda Hospital, Milan, Italy; ^2^Papa Giovanni XXIII Hospital, Bergamo, Italy; ^3^Azienda Sanitaria di Firenze, Florence, Italy; ^4^Mission Bambini ONG, Milan, Italy; ^5^Congenital Cardiac Services, University Hospital Southampton NHS Foundation Trust, Southampton, United Kingdom

**Keywords:** pediatric, children, heart, surgery, LMICs, humanitarian

## Abstract

**Background:** Every year, around 15 million children, in developing countries, die or develop life-long disabilities because of congenital cardiac diseases. In this report we measure the effect of a pediatric cardiac surgery humanitarian project on the health of the individual and on the potential influence this has on the countries economy and its growing health services.

**Methods:** We collected and analyzed data from the Italian NGO, Mission Bambini's database, including all congenital cardiac missions undertaken in Cambodia between 2012 and 2019. DALY's (Disability Adjusted Life Years) saved by the humanitarian mission were estimated and used to reflect on the impact this had on the populations economy. Progression in the local medical teams skills emulated the advancements made in the health sector of the region.

**Results:** Between 2012 and 2019, 128 patients underwent a congenital cardiac operation at Angkor Hospital for Children at Siem Reap, Cambodia. The median age was 6 years. The majority of the pathologies included VSD, TOF, ASD. The mean Aristotle's Complexity Score was 6. Post-operative mortality was 0.8% (1/128). The cost-effectiveness analysis identified 5.360 DALY's saved by surgery. The competency of the local team was progressive with them being able to handle more complex cases on subsequent missions.

**Conclusion:** In developing Countries, performing congenital cardiac surgery cases can be carried out successfully with improvement in both the economy and the health system of the country by increasing the years and the quality of life of the working population and developing the expertise of the regional team.

## Introduction

The majority of congenital heart conditions can be treated safely in more advanced healthcare systems, and more than 90% of these patients reach adulthood. Such progress has not been seen in low- to medium-income countries where the majority of patients who survive their underlying cardiac conditions carry with them a great burden of disability. To quantify the effects of disease, injuries, and risk factors on the health of the world's population, the notion of Disability Adjusted Life Years (DALY) has been introduced (1). The DALY can measure the burden of disease in terms of years of life lost (YLL) and years lost due to disability (YLD). One DALY corresponds to 1 year of health lost due to disability or early death. The Global Burden of Disease study reports a severity of 361 DALYs per 1,000 habitants at a global level (2), while congenital anomalies are responsible for over 120 DALYs per 1,000 children (3). Taking into account the number of children with congenital cardiac malformations in ratio to the size of the working population (4), it is evident that treating these malformations consequently leads to the economic growth of low-income countries.

The most commonly treatable congenital heart malformations (i.e., tetralogy of Fallot, ventricular and atrial septal defects) are responsible for an estimated 58% of preventable (in countries with advanced cardiac surgery services) diseases, which carries with it an overall preventable 38.9 million DALYs per year. The preventable deaths, from congenital cardiac pathologies, are ~66%, giving a total of 388,000 preventable deaths annually.

In low-income countries, treatment of congenital anomalies, particularly congenital cardiac anomalies, is undertaken predominantly by nongovernmental organizations (5).

The model of interventions and cooperation, followed by NGOs like Mission Bambini, predicts the development of a screening and early diagnosis program formed by the local cardiologists and the essential equipment supplied by the healthcare service of the territory (6–9). Once the conditions that carry the greatest number of years lost per disability (YLD) have been identified, it is possible to pinpoint the widest margin of preventable DALYs through targeted interventions weighted against the cost of each intervention.

**Figure 1:**
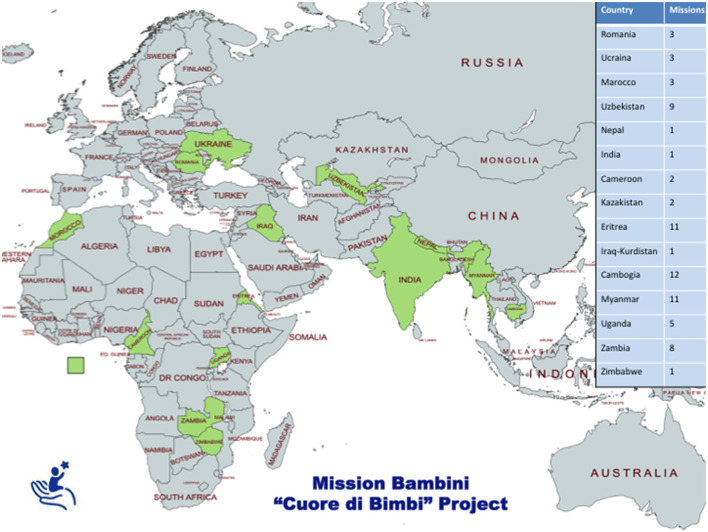
Mission Bambini Cuore di Bimbi projects.

The umbrella name of the humanitarian organization is called Bambini, an organization that deals with various needs of children in developing countries. Mission Bambini raises funds through charity drives and donations. The staff undertaking the missions are voluntary and unpaid. The project handling congenital heart disease is called “Cuore di Bimbi” (Children's Heart). This project was started in 2005 and has been implemented in 14 low-income countries until 2019. So far, 73 missions have been performed, with a total of 710 patients being treated (Figure 1). The aim of “Cuore di Bimbi” is to treat patients and teach the local services management of heart disease with the ultimate aim being their independence. Initial contact is made by the local hospital asking for assistance with congenital cardiac disease. A preliminary mission is undertaken to ascertain if the setting is suitable. The essential requirements are as follows: two pediatric ITU beds, one operating room, one cardiopulmonary bypass machine, one local surgeon (not necessarily cardiac), a local cardiologist, one intensivist, and nursing staff. If all requirements are met on the preliminary mission, the subsequent mission will be operative and teaching.

The selection of patients depends on the logistical means, financial resources, and the clinical skills of the local healthcare system, i.e., their diagnostic skills both antenatal and postnatal, the efficiency of the intensive care unit including the neonatal ITU, the availability of an ECMO program, and the permanent presence of a cardiologist and intensivist that would ensure the well-being of patients long after the mission has been completed. It is important to recognize that the sustainability of each project depends on the host country, and thus, patients are selected in keeping with their local context. Therefore, the degree of complexity treated is different for each project, but overall, the Aristotle Score is medium at the most. A mission is considered accomplished once the local unit is able to independently manage children with low- to medium-complexity congenital cardiac disease.

## Materials and Methods

In this study, we collected data from the NGO Missions Bambini's database, including data of 128 patients, mainly children, operated between 2012 and 2019 in Cambodia. The data included the following: demographic data, diagnosis, surgical interventions and outcomes, as well as financial data from the NGO including all costs incurred on each trip. The diagnosis was not clearly evident in 14 children, and they were thus excluded from the study, leaving a total of 114 children in the cohort. Demographic data used to estimate the DALY included the life expectancy of the population of Cambodia, the disability weight of various congenital cardiac pathologies, and their life expectancy if untreated, which were recovered from the WHO website and other medical literature (Appendix). The competencies of the local team were recorded on each mission, and greater challenges were introduced as appropriate. Analysis of the data was done using Microsoft Excel 2010.

## Results

“Cuore di Bimbi” has undertaken missions in Cambodia for 7 years, between 2012–2016 and 2018–2019. The average duration of each mission was 5 days. A total of 128 patients were screened. Out of the total, 14 did not have indication for surgical treatment; therefore, 114 patients, who underwent congenital cardiac surgical intervention, were enrolled into the study. The median age was 8 years: three were infants. Among the patients, 17% were between 0 and 1 year of age. The median weight was 16 kg (range 2.9–56 kg). The congenital cardiac malformations were 53 ventricular septal defects (VSDs), 31 tetralogy of Fallot (ToF), 21 atrial septal defects (ASDs), 1 total anomalous pulmonary veins defect (TAPVD), 2 sub-aortic stenosis (SAS), 2 mitral regurgitation (MR), 3 AVCa 3, and 1 other (Table 1).

**Table 1:** Demographic data.

**Demographic data**
Number of Patients	114
Mean Age (Yrs)	8
Mean Weight (Kg)	16
**Diagnosis**
ASD	21
VSD	53
ToF	31
Subaortic Stenosis	2
Mitral Regurgitation	2
AVSD	3
TAPVD	1
Sinus of Valsalva Fistula	1

The average complexity score based on the Aristotle Scoring System was 6 (10) (level 2). The postoperative mortality was 0.8% (1/114). The total cost of the Missions in Cambodia, including travel fees, boarding, visa fees, travel insurance, and medical supplies, was 176,000 euros, with an average expenditure of 22,000 euros per year. The average expenditure per cardiac operation was 1,375 euros. DALY and cost effectiveness analysis: the total number of DALYs saved was 5,364. The total DALYs saved can further be broken down to view the DALYs saved per pathology. The average DALY saved for the tetralogy of Fallot, ASD, and VSD groups is 57, 48, and 41, respectively.

Advancements in the competencies of the local surgical team were recorded. The local senior surgeon was a general surgeon with no previous exposure to cardiac surgery. He assisted on all the cases. He performed 30 ASD closures: 20 under supervision and 10 independently; he also performed 20 VSD closures, 10 of which were done autonomously. He additionally undertook, under supervision, one supracardiac TAVPD and one sinus of Valsalva fistula correction. Finally, he performed three tetralogy of Fallot repairs, one of them in autonomy. Simultaneously, the medical and nursing staff showed a significant progression in and the early postoperative management both in the intensive care unit and on the ward.

## Discussion

Low-income countries face ongoing healthcare challenges. Health economy is directed toward the more common and less costly diseases, leaving numerous patients with uncommon conditions with little or no possibility of treatment. Congenital heart disease patients represent a group of patients who are undertreated in these circumstances. The impression is that dealing with congenital cardiac surgical diseases is too expensive and brings too little benefit to the population as a whole, leaving scarce investment in these patients. Implementing a program (NGO) to treat congenital cardiac diseases in developing countries provides a viable transient solution for these children, and as our results demonstrate, it is also beneficial to the economy and development of the healthcare infrastructure of that country.

**Figure 2:**
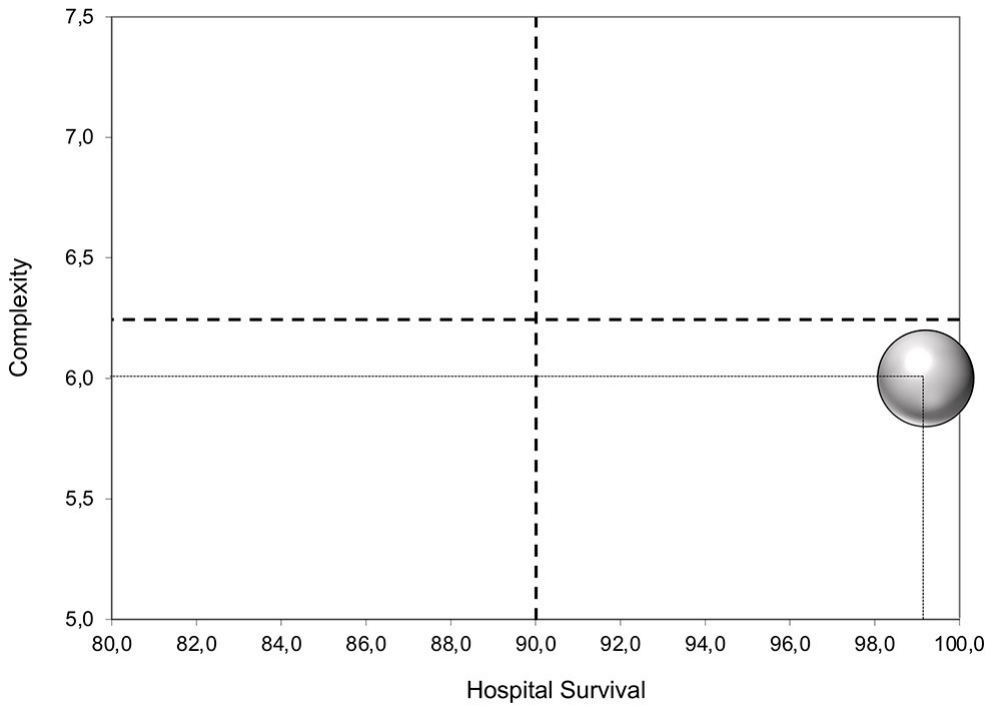
Surgical performance.

Establishing a program to treat congenital cardiac disease with remote and limited access to the patients is challenging. The ability to fully screen and prepare the patient preoperatively and to follow the patient through the entire postoperative course is impossible. Limited medical resources and skilled personnel are other factors to take into consideration. Despite these setbacks, with careful planning and effective collaboration with the local team, pediatric cardiac surgery can be done with favorable results. We have recorded an overall 0.8% mortality, which is favorable, compared with the literature on this topic (11). The complexity of surgeries was 6 according to the Aristotle risk score. Considering the ratio complexity/survival, our result is placed on the right-lower section of the performance evaluation of the Aristotle score (Figure 2) (10). The key factor in running a successful remote program is establishing a partnership with periodic missions to the same center. This gives a clear picture of the level of healthcare in the low-economic country, their limitations, and their potential for growth. This commitment fosters a trustful relationship for both parties in that the NGO can rely on the local teams' abilities to screen and prioritize patients, and the host team has the support of the NGO even remotely.

Performing congenital cardiac surgeries in low-economic countries through a charity mission is cost effective. In this study, the total cost of all the missions were 176,000 euros, and the total number of DALYs saved was 5,364. This is equivalent to 32.8 euro (total cost: DALYs = 176,000/5,364) per DALY saved. In other words, it costs 32.8 euros to give a person 1 year of life free of disability or death. As seen in Table 2, it is evident that the most total DALYs saved per pathology is in the VSD group; however, this is due to this being the commonest pathology treated. Of greater importance is the average DALY saved per patient, which is the highest among the tetralogy of Fallot group. It can be speculated that the more complex the disease, the higher the benefit in terms of DALY saved. These healthy years are spent either in education or at work, which contributes positively to the economy of the country. Of course, these missions are only financially viable because of donations and voluntary work undertaken by the medical teams. In the long run, one can hope that as a countries' economy improves, they would have more resources to invest in their healthcare with the eventuality of establishing an independent congenital cardiac treatment program. When this happens, the skills required to run this program would already be in the making.

**Table 2:** Average DALY's saved per pathology.

**Pathology**	**Num of patients**	**Tot DALY saved**	**Avrg DALY per patient saved**
VSD	53	2,168	41
ToF	31	1,834	57
ASD	21	1,024	48
Mix	9	336	42

The ultimate goal of any charity mission is to create independence of the host unit so that they could eventually help themselves. In this study, we can see that repeated missions to the same unit allow for the development of the skills in that unit. The local surgeon who only had training as a general surgeon with no cardiac background progressed to being able to perform simple operations like ASD repairs. In addition, invaluable skills were developed in the perioperative care of the congenital cardiac patient, evidenced in the hosts' ability to care for more complex patients independently during the latter years of the missions.

## Conclusion

Charity medical missions provide an invaluable humanitarian healthcare service in less wealthy areas of the world. Numerous children affected by correctable congenital heart diseases in those regions are denied treatments that are routinely performed in first-world countries. The reluctance of low-income countries in treating congenital heart disease lays into the impression of making a hopeless investment for the future. In our report, we have shown that performing medium-complexity congenital heart surgery in poor countries is feasible, with good results, and offers a solid contribution to the local economy by reducing the years of disability, which, in turn, has a direct impact on increasing the life expectancy of a population, improving education, and consequently improving the economy of a country as a whole.

## Data Availability Statement

The raw data supporting the conclusions of this article will be made available by the authors, without undue reservation.

## Author Contributions

SM, GC, NU, and SG contributed to conception and design of the study. SM organized the database. NU performed the statistical analysis. GC and SM wrote the first draft of the manuscript. CC, NV, and GA wrote sections of the manuscript. All authors contributed to manuscript revision, read, and approved the submitted version.

## Conflict of Interest

The authors declare that the research was conducted in the absence of any commercial or financial relationships that could be construed as a potential conflict of interest.

## Publisher's Note

All claims expressed in this article are solely those of the authors and do not necessarily represent those of their affiliated organizations, or those of the publisher, the editors and the reviewers. Any product that may be evaluated in this article, or claim that may be made by its manufacturer, is not guaranteed or endorsed by the publisher.
