# Supplementary material for: Analysis of a Cooperation and Interventional Model in Humanitarian Medicine
Source: Front Pediatr. 2021 Oct 28;9:705149. doi: 10.3389/fped.2021.705149 (PMC8582602; doi:10.3389/fped.2021.705149)
Supplement: Supplementary file 1 [file Data_Sheet_1.doc]

**Appendix**

**DALY**

In order to calculate the average savings of DALY’s (disability adjusted life years) per operation, we considered the years of life lost to disability and premature death for each type of congenital cardiac malformation (DALY’s per diagnosis) and the severity of the disability (disability weight) of each pathology derived from the study undertaken by Marcelo Cardarelli, et al. ^11^

The equation used to calculated the average savings of DALY’s (years of life lost to disability and premature death) for each patient is:

(f – {[a – (a x b)] – d}) – (f – {[a – (a x b)] – d} x h) = i

Where:

a = natural progression of the pathology

b = disability weight per compensated cardiac circulation (0.35 of the natural progress) (20)

a – (a x b) = c

c = a – (axb) = true life expectation per pathology in the absence of surgical intervention

Comment:

From the life expectancy of each congenital cardiac defect, you subtract the years of life lost to disability due to a decompensated cardiac circulation.

[f – (c – d)] – [f – (c – d) x h] = i

Where:

d = age of patient at the time of surgical intervention

e = c-d = life expectancy of a specific patient in the absence of cardiac intervention.

Comment:

The closer the age of the patient is to the true life expectancy of each specific diagnosis, the greater the impact surgical intervention has on the saved DALY’s, as is possible to observe in the following equation: (f – e) – ([f – e] – h) = i

Where:

f = average life expectancy per country of origin for each specific patient.

g = f-e = years of DALY’s saved by surgical intervention

i = g – (gxh)

Comment:

Geographical regions that have a greatly reduced life expectancy, inevitably adjust the weight of interventions in terms of DALY’s saved.

Where finally:

h = disability weight that each surgical intervention has on a specific congenital cardiac defect (table 1 above).

Comment:

From the DALY’s saved through surgical intervention, one can consider and hence subtract, the years of life lost to disability as a result of surgical intervention for each specific congenital cardiac defect.

This is the way to calculate the DALY’s saved by surgical intervention.

The following is reported in the life expectancy table for each congenital defect when surgical intervention if not available.

**Cost effectiveness analysis ^12-13^**

In order to calculate the average cost of surgery per saved DALY, we subtracted the total number of years of life lost mortality post surgery. The years of life lost to mortality post surgery are calculated from: *Natural history of pathology – Age of patient at time of surgical intervention.*

Once we have subtracted the years of life lost to mortality post operatively from the total DALY’s saved due to surgical intervention of the entire cohort, it is possible to proceed with the calculation of the average DALY’s saved for each patient operated.

With the knowledge of the average cost of each surgical intervention, we can derive the cost of each intervention for each year of life without mortality or premature death.

From the analysis of the cost effectiveness, it is possible to confront the true efficiency of the described interventional model in terms of DALY’s saved.

**A PATIENT EXAMPLE OF DALYs AVERTED**

**Fallot operated at 5 Years of Age**

**a**= 10 yrs ( natural history) **b**= 0,35 disability weight for end stage heart failure for untreated CHD (0.35 of the natural history)

**a-(a x b) = c**= 10-(10 x 0,35) = 6,5 years ( true life expectancy for that specific diagnosis without treatment)

and where:

**d**= 5 years at time of surgery

**c-d = e** = 6,5 years – 5 years = 1,5 years ( life expectancy for a specific patient with a specific diagnosis without treatment)

with:

**f**= 65.2 years ( life expectancy for Cambodia)

**f – e** **= g** 65,2 – 1,5 = 63,7 ( years of disability or early death avoided by surgery)

and were:

**h**= 0,20 DALYs lost even after treatment, due to need for future re- operation, complications of the surgery, progression of the disease, etc.

**g - (g x h)= i** 63,7- (63,7 x 0,20) =(63,7 – 12,7) = **51 DALYs averted for this patient.**
